# BCAM (basal cell adhesion molecule) protein expression in different tumor populations

**DOI:** 10.1007/s12672-024-01244-1

**Published:** 2024-08-29

**Authors:** Sneha Burela, Mengni He, Ioannis P. Trontzas, Niki Gavrielatou, Kurt A. Schalper, Solomon Langermann, Dallas B. Flies, David L. Rimm, Thazin N. Aung

**Affiliations:** 1 Department of Pathology, Yale School of Medicine, New Haven, CT 06519 USA; 2 School of Medicine, National and Kapodistrian University of Athens, Athens, Greece; 3 NextCure Inc, Beltsville, MD USA

**Keywords:** The basal cell adhesion molecule (BCAM), Quantitative immunofluorescence (QIF), Cancer biomarkers, PD-L1 expression, Therapeutic target

## Abstract

Basal Cell Adhesion Molecule (BCAM), a receptor for laminin subunit α5, plays a crucial role in the pathogenesis of various malignancies. Notably, evidence of hypermethylation at multiple immune checkpoints in patients with low BCAM expression suggests these individuals may respond favorably to immunotherapy using ICIs (immune checkpoint inhibitors). This finding lays the foundation for the hypothesis that BCAM may serve as an important biomarker in cancer patients. To investigate this potential, we evaluated BCAM expression patterns in 3114 patients from both discovery and validation cohorts, spanning seven cancer types, using quantitative immunofluorescence (QIF). We also explored the correlation between BCAM and PD-L1 expressions within these cohorts, aiming to establish its potential predictive value for immunotherapy response. Our findings indicate that BCAM was highly expressed in ovarian (79.2%) and lung (78.5%) tumors, with lower yet significant expression in breast (37.7%), head and neck (31.3%), and bladder-urothelial tumors (27.6%). Notably, high BCAM expression was associated with better OS in NSCLC. More importantly, BCAM expression did not correlate with PD-L1 protein expression in any of these tumors, highlighting its independent predictive potential. The widespread expression of BCAM across multiple tumor types, coupled with its lack of correlation with PD-L1 expression, highlights its potential as a predictive novel biomarker across various cancer types.

**Supplementary Information:**

The online version contains supplementary material available at 10.1007/s12672-024-01244-1.

## Introduction

Basal Cell Adhesion Molecule (BCAM) is a 90 kDa membrane-bound glycoprotein of the immunoglobulin superfamily, also known as the Lutheran blood group glycoprotein (Lu) or CD239. It is a receptor for the extracellular matrix protein laminin subunit α5 [1]. This protein has five extracellular immunoglobulin domains, a single transmembrane domain, and a short C-terminal cytoplasmic tail and is widely expressed in epithelial, endothelial, and other cell types [1]. BCAM is involved in the pathogenesis of several diseases, such as sickle cell anemia, polycythemia vera, and glomerulonephritis [2]. Moreover, there is ample evidence describing the association between BCAM and various cancers [2–5]. Overexpression of BCAM is observed in many epithelial cell cancers, such as ovarian carcinoma, skin cancer, breast cancer, colorectal cancer, and hepatocellular carcinoma [3–6].

Mechanistically, BCAM contributes to carcinogenesis by promoting cancer cell adhesion to basement membranes, cell chemotaxis, and tumor cell migration through the disruption of normal cell–cell and cell–matrix interactions; it is thus related to the invasion and metastasis of tumor cells [3]. It is believed that BCAM plays a role in tumorigenesis through the modulation of integrins binding to the laminin α5 chain, which is a major component of the basement membrane [7]. BCAM competes with integrins and through the preferential binding of BCAM to laminin, tumor cell migration is promoted [8]. A laminin-associated tumorigenesis mechanism has been described in the Erk-related signaling pathway of Lu/BCAM [9]. Lu/BCAM binds to laminin-10/11 (subunit LAMA 5) and activates the Erk signaling pathways, which play an active role in tumorigenesis [10].

It has also been reported that high levels of BCAM in renal, pelvic, ureteral, and bladder cancers are significantly associated with advanced tumor stage, larger tumor size, and lower disease-specific survival [9]. This information provides insight into the nature of BCAM as a potential biomarker of interest and therapeutic candidate.

A previous study on clear cell renal cell carcinoma showed that multiple immune checkpoints, including CD274, CTLA4, HAVCR2, LAG3, PDCD1, PDCD1LG2, and TIGIT, were overexpressed in patients with low BCAM expression. Furthermore, these immune checkpoints were found to have lower methylation levels in the BCAM-low subgroup than in the BCAM-high subgroup, highlighting that the former might respond better to immune checkpoint inhibitors (ICIs) therapy [11]. Immune therapies, such as the ICIs, have been successfully used to treat many patients with advanced solid tumors. Although ICIs have many applications in the treatment of solid tumors, there are currently grey areas that lead to low efficacy of these drugs in many patients. The limitations of the current therapies highlight the need to investigate new potential targets, such as BCAM, which can be targeted alone or in combination with the PD-1/PD-L1 axis [7, 12, 13].

Here, we aimed to assess the expression of the BCAM protein in several cancer types, with the potential to develop a standardized companion diagnostic assay for BCAM-targeted therapy. To achieve this goal, we screened seven cancer types from 12 different cohorts having 3114 patient samples in total, using quantitative immunofluorescence (QIF) techniques. Moreover, the prognostic importance of BCAM has been explored in these types of cancer. This study also evaluated the correlation between BCAM and PD-L1 expression as a surrogate for potential dual therapeutic targeting.

## Materials and methods

### Study population

Tissue biopsies from 3,114 patients were retrospectively collected, and representative tumor specimens were selected and sectioned into 0.6 mm cores for incorporation into tissue microarrays (TMA) using standard procedures [14]. All tissue samples were collected with approval from the Yale Human Investigation Committee protocol #9505008219. Written informed consent or a waiver of consent was obtained from all patients with the approval of the Yale Human Investigation Committee. The following TMA cohorts were studied- (1) one multi-tumor array, YTMA (Yale TMA)-395 (containing 235 cores of 13 different tumors retrieved from year 1997–2015), (2) two NSCLC (non-small cell lung cancer) arrays, YTMA-423 and YTMA-553 (containing 287 and 296 cases retrieved from year 2011–2016 and 2017–2020, respectively), (3) two ovarian carcinoma arrays, YTMA-69 and YTMA-264 (containing 358 and 335 cases retrieved from year 1996–2003 and 1963–2001, respectively), (4) two CRC (colorectal carcinoma) arrays, YTMA-221 and YTMA-410 (containing 254 and 151 cases retrieved from year 2000–2005 and 2009–2017, respectively), (5) two breast carcinoma arrays, YTMA-489 and YTMA-499 (containing 263 and 183 cases retrieved from year 2011–2012 and 2013–2014, respectively), (6) two bladder carcinoma arrays, YTMA-361 and 497 (containing 230 and 42 cases retrieved from year 2005–2014 and 2015–2019, respectively), (7) two head and neck squamous cell carcinoma (HNSCC) arrays YTMA-465 and 579 (containing 204 and 247 cases retrieved from year 1998–2018, and 2019–2021, respectively), (8) one pancreatic carcinoma array, YTMA-454 (containing 238 cases retrieved from year 2010–2017) (Supplementary Table 1). The clinicopathological characteristics of each cohort are presented in supplementary Tables 2–7. A minimum of three slides of the above-mentioned tumor cohorts were stained using a QIF protocol (two serial tissue sections from the same block of TMA and a separate section from a different block to make it two-fold redundant) to assess the reproducibility and BCAM expression heterogeneity within the tumor. An index TMA (YTMA-558) with BCAM-positive and-negative cell line controls and a few tumor cores, including ovarian cancer, lung cancer (NSCLC), and CRC cases, was constructed for antibody validation and optimization purposes.

### Antibody validation

Five commercially available BCAM antibodies and five biotinylated antibodies provided by NextCure Inc. (10 antibodies in total) were used. The signal-to-noise ratio (SNR) was used as a measure of the QIF signal for each tested antibody. The antibody with the highest SNR was selected for further evaluation of BCAM protein expression in different tumor populations. Monoclonal anti-human BCAM antibodies included AB111181 (Abcam Inc., Cambridge, Massachusetts, USA), MCA 1982 (Bio-Rad Laboratories Inc., Hercules, California, USA), MM0107 (Novus Biologicals, LLC, Colorado, USA), and FQS5276 (Creative Diagnostics, Shirley, New York, USA). The polyclonal antibody HPA005654 (Sigma Aldrich, Inc., St. Louis, Missouri, USA) along with biotinylated antibodies NP095, NP638, NP639, NP640, and NP641 (Next Cure Inc., Maryland, USA) were also tested. The details of these antibodies are provided in the Supplementary Information. The optimal working concentration of each antibody was determined by titration using a log_2_ range serial dilution for each antibody. The antibody concentration with the highest SNR in the index array YTMA-558 was considered optimal. Antibody validation for protein expression was performed according to the previously formulated standards [15].

### Quantitative immunofluorescence (QIF)

The protocol for the QIF assay, including sample preparation, blocking, primary antibody incubation, secondary antibody incubation, and imaging, has been previously documented [15]. Briefly, TMA slides were deparaffinized in xylene and rehydrated in graded ethanol baths. Heat-induced antigen retrieval was performed using a citrate buffer (pH 6.0) at 97 ℃ for 20 min. Permeabilization and blocking of endogenous peroxidases were performed by incubating the slides for 30 min in 0.3% hydrogen peroxide in methanol and then in a blocking solution of 0.3% bovine serum albumin and 0.05% tween-20 for 30 min [15]. Tissue slides were then incubated at room temperature for 1 h with a primary antibody mixture consisting of anti-BCAM (AB111181), which was the target antibody, and anti-cytokeratin (clone AE1/AE3, Dako), an epithelial marker that was used to mark the tumor area later for analysis, followed by a secondary antibody mixture consisting of anti-rabbit Envision (Dako) and anti-mouse Alexa Fluor 546 (Invitrogen). Subsequently, tyramide cyanine 5 (PerkinElmer) was used for signal amplification, followed by nuclear staining with DAPI (4′,6-diamidino-2-phenylindole). The slides were rinsed in TBST and TBS buffer before and after incubation. Prolonged gold antifade reagent (Invitrogen) was used for slide mounting. A more detailed description of the staining protocol is provided in Supplementary Table 8.

### PD-L1 staining

TMA slides from five tumor types, including ovarian, lung (NSCLC), breast, bladder, and HNSCC, were stained for PD-L1 using E1L3N clone, a rabbit monoclonal antibody (Cell Signaling Technology, CST, Massachusetts, USA), following the above-mentioned QIF staining protocol. Antigen retrieval was performed at pH 8.0. using ethylenediaminetetraacetic acid (EDTA) buffer. The optimal concentration of the antibody used was 1.1 µg/ml as previously established in a standardized staining protocol [16]. Details of the staining protocol are provided in the (supplementary information Table 9).

### Image acquisition and analysis

AQUA (Automated Quantitative Analysis) version 3.0, a software tool for digital pathology, was used to measure BCAM protein expression on fluorescence images acquired using a PM-2000 microscope system (Navigate BioPharma) according to standard operating procedure [17]. The total compartment of all cells generated by thresholding the DAPI signal. Target antibody-BCAM was measured within the tumor mask/compartment defined by masking cytokeratin generated in Alexa 546 channel. Stromal compartment was created by excluding the tumor compartment from the total. Expression of the protein in tumor tissues was measured based on AQUA scores generated for each protein by dividing the sum of target pixel staining intensities by the area of the designated compartment. The final value or measurement of signal/staining intensity of the antibody in tumor compartment was calculated by averaging the AQUA scores generated for each tumor spot in the three slides (two serial sections cut from same block of the tumor and a separate section/slide cut from another block of the TMA). Exclusion criteria of tissues from analysis were spots with inadequate tumor tissue, missing spots/exhausted tissue, or artifacts such as tissue folding and air bubbles. Cases with no clinical information were excluded from survival outcome analysis as well.

### Statistical analysis

Statistical analyses were performed using GraphPad Prism 9.2.0 (GraphPad Software Inc., CA, USA). For all tumor types and cohorts used in this study, we estimated the prognostic value of BCAM using the Kaplan–Meier product-limit method and compared it using the log-rank test in GraphPad Prism. We used the median as the cut-off to divide the BCAM-expressing tumors into two groups (Low BCAM group ≤ median, High BCAM group > median). We also established a visual cut-off point for positive BCAM-stained cases by inspecting all cases and selecting the one where minimal membranous staining could be recognized for each cohort. This allowed for the characterization of the proportion of positive BCAM cases and was used as an alternative cutoff point. Comparisons were performed using the log-rank test. All statistical tests were two-sided, with a level of significance < 0.05. A linear regression model was used to compare the correlation coefficient (R^2^ value) between the expression of PD-L1 and BCAM, as well as to plot the reproducibility between the serial sections and intratumoral heterogeneity.

## Results

### Antibody validation

**Fig. 1:**
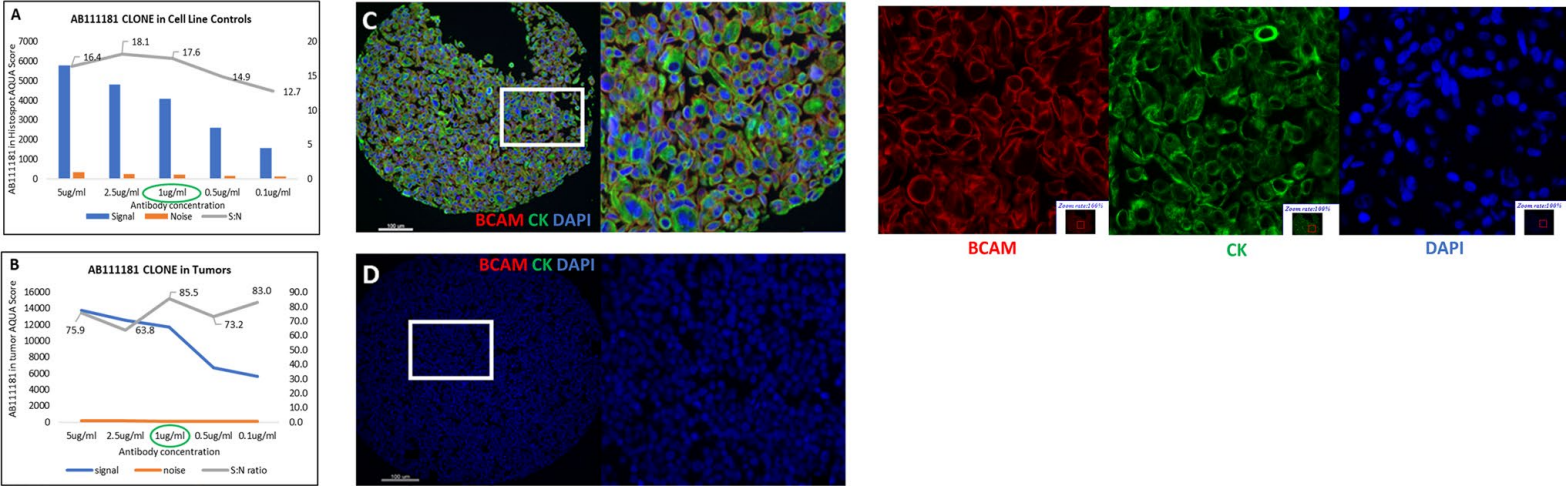
Antibody validation. **A** Antibody validation in BCAM index array YTMA-558. Signal to noise ratio of BCAM antibody- AB111181 in positive and negative cell line controls. **B** Signal to noise ratio of BCAM antibody- AB111181 in tumors. **C** Representative low and high-power merged images and monochrome single channel of positive cell line control (BEWO). **D** Representative high and low power merged images of negative cell line control (JURKAT). Merged Image- BCAM (Red), Cytokeratin (Green), DAPI (Blue)

BCAM index array YTMA-558 was constructed for antibody validation. In cell line controls with positive or negative BCAM expression, signal was calculated as average AQUA score (staining intensity) of the positive cell line controls and noise was calculated as average AQUA score of the negative cell line controls. The SNR for tumors was calculated by using the average AQUA score of the 10% of the spots with the highest BCAM expression to determine the signal and the average AQUA score of the 10% of the spots with the lowest BCAM expression to determine the noise. (Supplementary Fig. S1). Out of all the monoclonal antibody clones tested for different epitopes of the antigen, AB111181 showed a wide dynamic range of signal or staining pattern on the cell membranes and the SNR peak was at the concentration of 1 µg/ml (microgram per milliliter) of the antibody for both cell line controls and tumor tissues (Fig. 1). Other antibodies including the polyclonal biotinylated antibodies did not validate in the cell line controls tested (Supplementary Fig. S1). Thus, we continued further QIF staining on larger tumor type-specific cohorts with the AB111181 clone.

### Target population screening—tumor types expressing BCAM protein

**Fig. 2:**
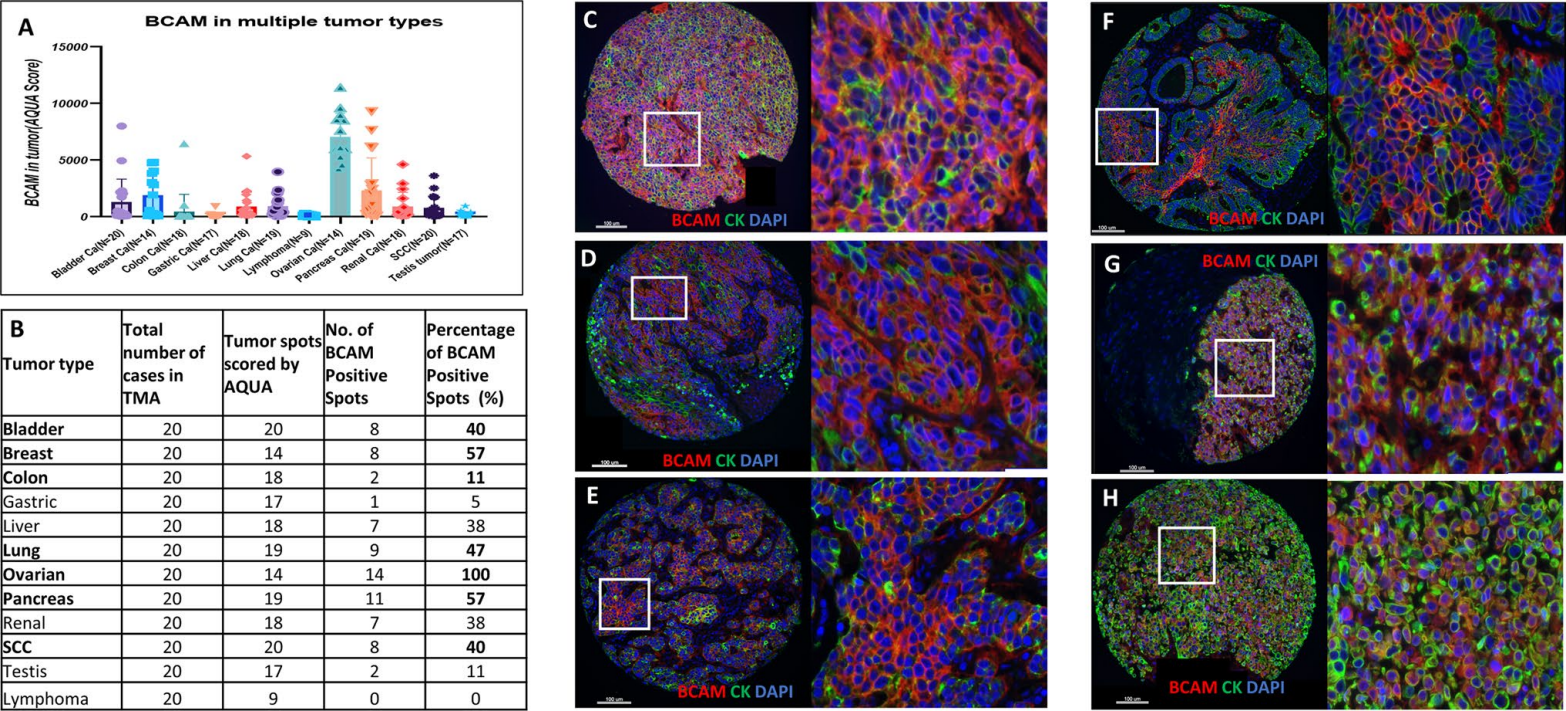
Target population screening—tumor types expressing BCAM protein. **A** BCAM antibody- AB111181 in various tumors multi tumor array YTMA-395. **B** Expression levels of BCAM in each tumor. Representative high and low power images of tumors (**C**) Ovarian carcinoma (**D**) Lung carcinoma, **E** Breast carcinoma, **F** Colorectal Carcinoma, **G** Pancreatic carcinoma, and **H** Bladder Carcinoma. Merged Image-BCAM (Red), Cytokeratin (Green), DAPI (Blue)

After antibody validation, we performed the screening of BCAM expression in several tumor types. A broad range of BCAM protein expression measured by signal/staining intensity in QIF was seen across multiple tumor types in YTMA-395 (multi-tumor array). 203 out of the 235 tumor cores were studied (32 tumor tissue spots were excluded from analysis due to exhausted tumor tissue) including breast, bladder, CRC, gastric, liver, lung (NSCLC), ovarian, pancreatic, renal, testicular, squamous cell carcinomas, and lymphomas. The higher levels of BCAM protein expression were seen in ovarian, lung (NSCLC), breast, bladder, pancreatic and squamous cell carcinoma tissues (Fig. 2). Based on this observation, we further explored the staining pattern of BCAM in these specific tumor types in larger tumor population for each type. We noticed a membranous pattern of staining with the BCAM antibody in the tumor cells for all the above-mentioned cancer types.

### Expression of BCAM protein in ovarian cancer and correlation with PD-L1

**Fig. 3:**
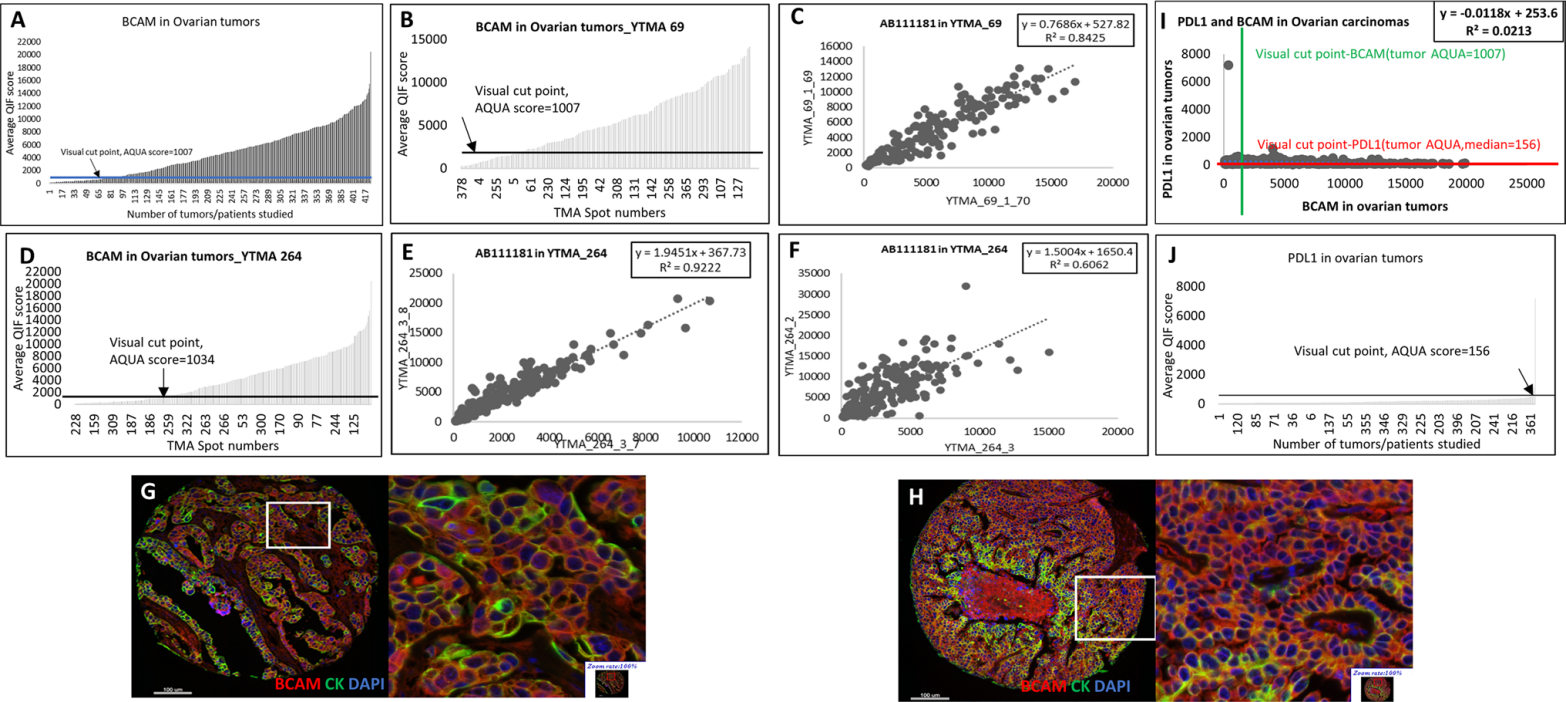
BCAM expression in ovarian tumors. Dynamic range of BCAM expression levels in (**A**) all Ovarian tumor, **B** YTMA-69 and (**D**) YTMA-264. **C** Regression between the serial sections in YTMA-69 to check reproducibility. **E** Regression between the serial sections in YTMA-264 to check reproducibility. **F** Regression between different blocks in YTMA-264 to check intra-tumoral heterogeneity. **G** and **H** Representative high and low power images of tumors. **I** Regression between PD-L1 and BCAM in ovarian carcinomas. **J** Dynamic range of PD-L1 expression in tumors from both cohorts-YTMA-69 and -264. Merged Image- BCAM (Red), Cytokeratin (Green), DAPI (Blue)

Ovarian carcinomas with varied histologic types showed prominent levels of BCAM expression in two ovarian tumor cohorts that were studied. Expression of BCAM in ovarian tumors had a broad dynamic range with 79.2% of tumors/cases being above the visual threshold (n = 336/424 were BCAM positive). Regression analysis for the BCAM expression pattern between serial sections and between blocks of the same cohort demonstrated good assay reproducibility (R^2^ = 0.922 and R^2^ = 0.659, respectively) in YTMA-264. In YTMA-69, the R^2^ was 0.843 between the serial sections of the same block. Prognostic value of BCAM expression for ovarian cancer was tested in association with survival outcomes of the cohorts. No statistically significant association of BCAM expression with overall survival (OS) using either median cut point (p = 0.391; HR = 1.170) or visual cut point (p = 0.727; HR = 1.078) was observed. Survival outcome was plotted only for YTMA-264 (N = 196) as there was no clinical information available on YTMA-69 (Supplementary Fig. S4). We also examined the PD-L1 expression for 398 ovarian tumors and compared it with BCAM expression. The results showed no correlation between the two antibodies, with an R^2^ value of 0.021 (Fig. 3). The high expression of BCAM antibody in ovarian carcinomas is not significantly associated with prognosis and is nearly mutually exclusive with PD-L1, which is infrequently expressed, or expressed at relatively low levels in ovarian carcinomas.

### Expression of BCAM protein in non-small cell lung cancer and correlation with PD-L1

**Fig. 4:**
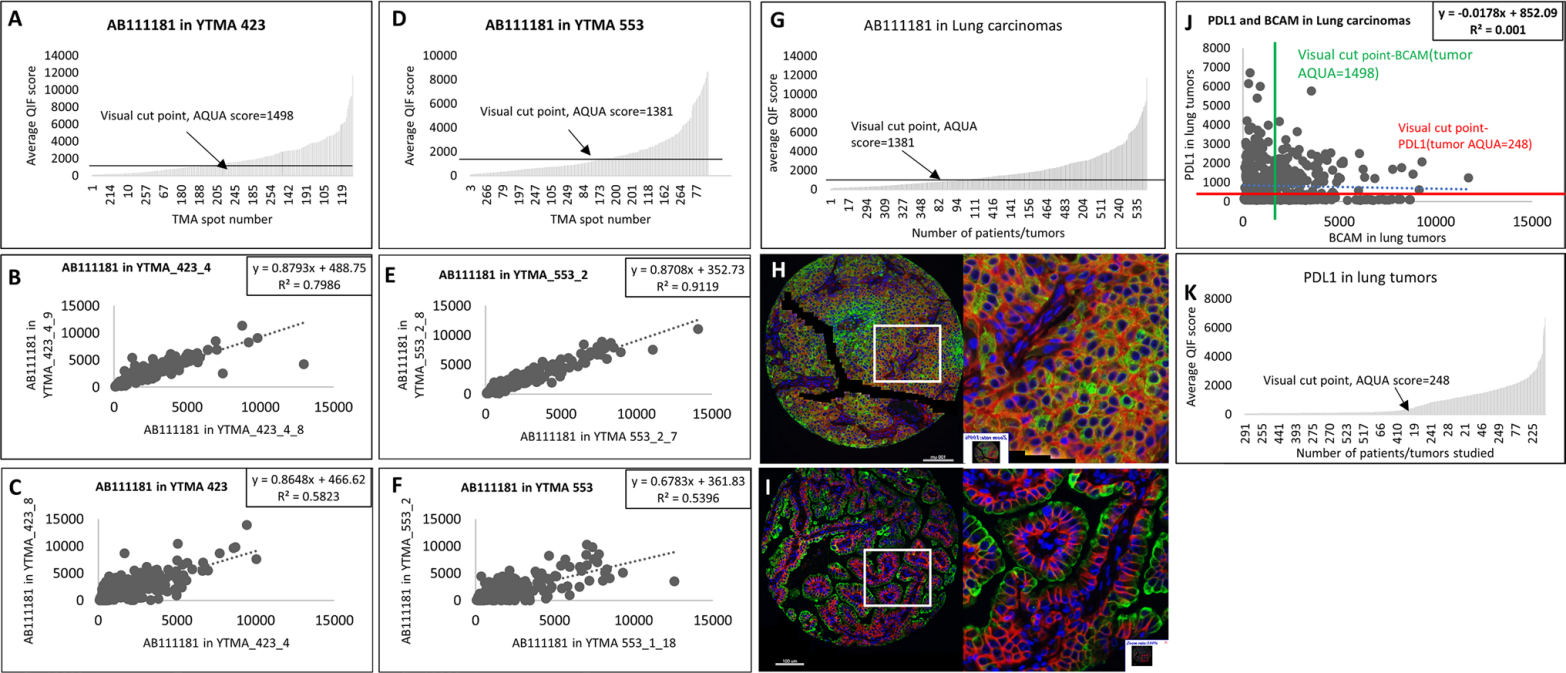
BCAM expression in lung tumors. Dynamic range of BCAM expression levels in (**A**) YTMA-423 (**D**) YTMA-553 and (**G**) tumors of both cohorts. Regression between the serial sections in (**B**) YTMA 423 and (**E**) YTMA 553. Regression between different blocks in (**C**) YTMA-423 and (**F**) YTMA-553. **H** and **I** Representative merged high and low power images. **J** Regression between PD-L1 and BCAM in lung carcinomas. **K** Dynamic range of PD-L1 expression in tumors from both cohorts-YTMA-423 and -553. Merged Image- BCAM (Red), Cytokeratin (Green), DAPI (Blue)

In lung cancer (NSCLC), 78.5% of the population (n = 281/545 cases) showed visible BCAM expression signal (positive cases). Serial tissue sections of the two cohorts, YTMA-423 and YTMA-553, demonstrated good assay reproducibility (R^2^ = 0.799 and 0.912, respectively). Comparison of BCAM expression between different blocks of the same cohorts revealed slightly diminished reproducibility (R^2^ = 0.582 and 0.54 for YTMA-423 and YTMA-553, respectively), probably accounting for intra-tumoral heterogeneity (Fig. 4). Notably, high BCAM expression was associated with better OS in NSCLC patients using both median cut point (p = 0.009; HR = 0.59) and visual cut point (p = 0.003; HR = 0.59) (Supplementary Fig. S4). In addition, BCAM expression was not correlated with PD-L1 expression in NSCLC subgroup (R^2^ = 0.001).

### Expression of BCAM in breast cancer and correlation with PD-L1

**Fig. 5:**
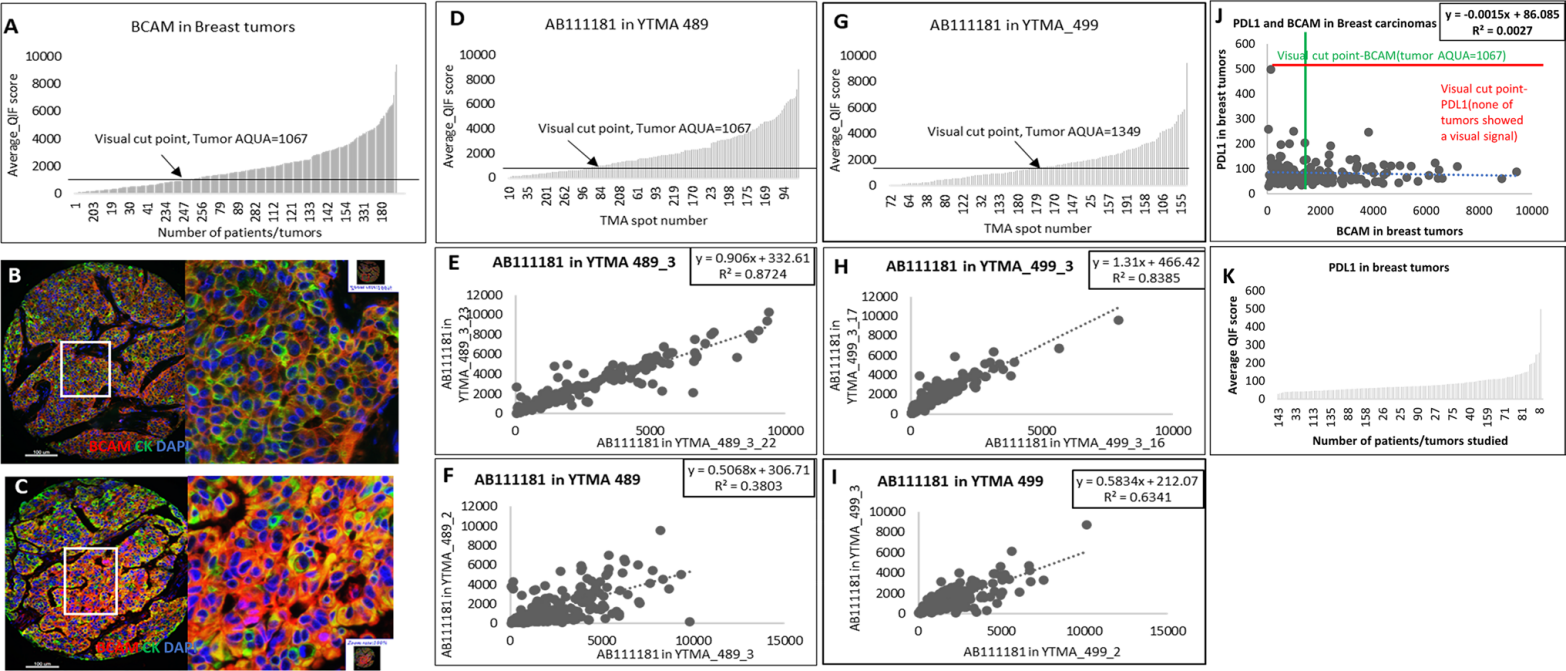
BCAM expression in breast tumors. Dynamic range of BCAM expression levels in tumors from (**A**) both cohorts, (**D**) in YTMA-489 and (**G**) YTMA-499. Regression between the serial sections in (**E**) YTMA-489 and (**H**) YTMA-499. Regression between different blocks in (**F**) YTMA-489 and (**I**) YTMA-499. **B** and **C** Representative high and low power images. **J** Regression between PD-L1 and BCAM in breast carcinomas. **K** Dynamic range of PD-L1 expression in tumors from both cohorts-YTMA-489 and -499. Merged Image- BCAM (Red), Cytokeratin (Green), DAPI (Blue)

Breast cancer patients demonstrated lower BCAM expression, compared to ovarian cancer and lung cancer (NSCLC). In particular, 37.7% (n = 128/339) were cases above the established visual threshold. Serial tissue sections of the two cohorts, YTMA-489 and YTMA-499, showed high intra-block reproducibility (R^2^ = 0.872 0.839, respectively). When staining different blocks to test for intra-tumoral heterogeneity regression dropped to R^2^ = 0.38 and 0.634 for YTMA-489 and YTMA-499, respectively (Fig. 5). BCAM expression was not prognostic for OS in breast cancer patients either using median cut point (p = 0.571; HR = 0.811) or visual cut point (p = 0.158; HR = 0.709) (Supplementary Fig. S4). In breast cancer cases (168 in total), BCAM expression did not show any significant correlation with PD-L1 expression, like ovarian and lung cancers (Fig. 5).

### Expression of BCAM in HNSCC and correlation with PD-L1

**Fig. 6:**
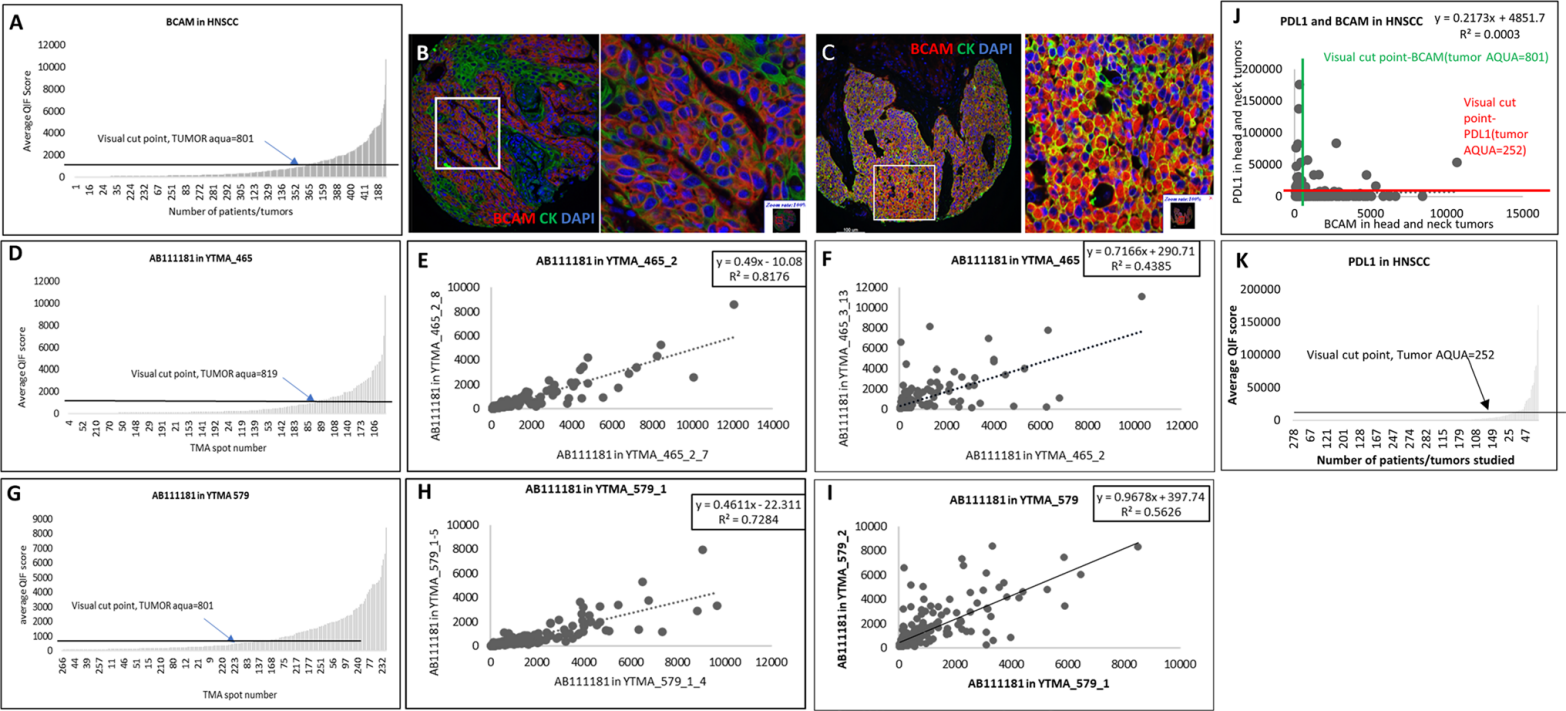
BCAM expression in HNSCC tumors. Dynamic range of BCAM expression levels in tumors from (**A**) both cohorts, in (**D**) YTMA-465 and (**G**) YTMA-579. **B** and **C** Representative high and low power images of tumors. Regression between the serial sections in **E** YTMA-465 and (**H**) YTMA-579. Regression between different blocks in (**F**) YTMA-465 and (**I**) YTMA-579. **J** Regression between PD-L1 and BCAM in HNSCC. **K** Dynamic range of PD-L1 expression in tumors from both cohorts-YTMA-465 and -579. Merged Image- BCAM (Red), Cytokeratin (Green), DAPI (Blue)

Two cohorts of HNSCC cases (n = 430), YTMA-465 and YTMA-579, including oropharyngeal, hypopharyngeal, and laryngeal carcinoma, were assessed for BCAM expression. In total, 31.3% (n = 135/430) were above the visual threshold for BCAM staining. Serial sections of the two cohorts showed high concordance (R^2^ = 0.818 and 0.728 for YTMA-465 and YTMA-579, respectively) in terms of BCAM expression. Different blocks of the same cohort were tested for BCAM expression, and they demonstrated lower concordance (R^2^ = 0.439 0.563 for YTMA-465 and YTMA-579, respectively), suggesting the influence of intra-tumoral heterogeneity (Fig. 6). BCAM expression was not significant for OS in both cut points, (p = 0.379; HR = 1.154 for median cut point) and (p = 0.509; HR = 1.128 for visual cut point) (Supplementary Fig. S4). In 282 cases of HNSCC, BCAM expression was unrelated to PD-L1 expression (R^2^ = 0.0003) (Fig. 6).

### Expression of BCAM in bladder *cancer* and correlation with PD-L1

Positive BCAM cases (above visual threshold) were comparatively low (27.6%, n = 54/195) in the two bladder cancer cohorts tested (YTMA-497 and YTMA-361). Serial sections staining provided high degree of reproducibility for both cohorts (R^2^ = 0.879 and 0.819 for YTMA-497 and YTMA-361, respectively). No correlation between BCAM and PD-L1 expression was seen in the bladder cancer cohort (R^2^ = 0.008) (Supplementary Fig. S2). Inter-block comparison for YTMA-361 provided high intra-tumoral heterogeneity (R^2^ = 0.40). BCAM was not prognostic for OS in bladder cancer patients with available clinical information, (p = 0.215; HR = 0.795 for median cut point) and (p = 0.891; HR = 0.971 for visual cut point) (Supplementary Fig. S4).

### Expression of BCAM in colorectal and pancreatic cancer and correlation with PD-L1

In CRC cohorts (YTMA-221 and YTMA-410), only 2.5% (n = 9/360) of cases showed BCAM expression above the visual threshold (Supplementary Fig. S3). KRAS mutant CRC cases showed minimal to no expression of BCAM and there was no significant correlation when KRAS mutation and BCAM expression were compared. Similarly, BCAM was not expressed (0.8%, n = 2/223) in the pancreatic cancer cases (YTMA-454) (Supplementary Fig. S3). Consequently, further studies were not pursued on these two tumor subgroups.

## Discussion

BCAM expression has been studied in several cancer types including ovarian, skin, lung, gastric, liver, thyroid, breast, and bladder cancer [10, 18–25]. In our study, we observed that ovarian carcinoma cases had high levels of BCAM expression, which did not correlate with PD-L1 expression since PD-L1 generally tends to be expressed at very low levels in this cancer type. The expression of BCAM RNA was associated with an unfavorable clinical outcome and pro-metastatic function, as stated by Siva Kumar et al. [18]. Although we were unable to confirm these findings with protein expression. The interaction of BCAM with its ligand, laminin α5, regulates cell adhesion, migration, and proliferation, primarily through the N-terminal immunoglobulin-like domains. The BCAM-laminin interaction activates signaling pathways that promote tumor cell motility and invasion, highlighting BCAM's potential involvement in tumor progression. This mechanism might explain the pro-metastatic function associated with BCAM RNA expression in cancer and other malignancies.

In NSCLC patients, our findings revealed that those with higher BCAM expression levels, as indicated by the average AQUA score, had improved overall survival when the population was divided into two groups using the median cut point and the visual threshold. No significant differences were observed when considering the histologic type of tumor (adenocarcinoma or squamous cell carcinoma) or any other clinicopathological parameters. Additionally, there was no correlation with PD-L1 expression. This contradiction highlights the context-dependent nature of BCAM’s prognostic significance, which may differ based on tumor microenvironment or therapy responsiveness. Importantly, the lack of correlation between BCAM and PD-L1 expression across cancer types indicates BCAM as an alternative prognostic marker and therapeutic target for patients less likely to benefit from PD-1/PD-L1 inhibitors.

The expression of BCAM in breast cancer patients indicates that it is a promising target for therapies such as antibody–drug conjugates (ADCs). Kikkawa et al. previously demonstrated that BCAM antibodies can be internalized into breast cancer cells, facilitating their conjugation with highly toxic agents, such as radioisotopes or systemic toxins, to develop ADCs [19]. The combination of BCAM monoclonal antibodies with the potent cytotoxic effects of small molecule drugs in ADCs might have their potential as a promising category of cancer therapeutics through precise targeting. Furthermore, ADCs targeting BCAM could circumvent drug resistance mechanisms, as their unique delivery method ensures that cytotoxic agents reach cancer cells directly. This versatility allows for the combination of BCAM-targeted ADCs with other therapeutic agents, providing a robust platform to tailor treatments for different BCAM-expressing cancer subtypes.

According to Schon et al. BCAM plays a significant role in keratinocyte proliferation and is highly expressed in squamous cell carcinomas [25]. Concordantly, we observed a similar pattern of BCAM expression in head and neck squamous cell carcinomas. In previous studies, it was described that BCAM was expressed with a basal pattern in the epithelium of the normal colon, prostate, ovary, and thyroid [26, 27]. This broad expression pattern underscores BCAM’s potential as a target in a variety of epithelial malignancies. Moreover, its significant role in tumor cell proliferation and invasion suggests that inhibiting BCAM may impair cancer progression and metastasis, establishing it as a promising candidate for targeted therapy.

This study faces several limitations. First, its retrospective design may lead to less accurate measurements of risk factors and outcomes. Second, the use of TMAs instead of whole tissue sections restricts the examined tumor area, potentially missing focal areas of heterogeneous expression. Third, the loss or exhaustion of some tumor tissues led to their exclusion from analysis, reducing the sample size and possibly affecting statistical power. Fourth, limited clinical information for certain tumor types, such as ovarian, bladder, and colorectal tumors, restricted the scope of our study by preventing comprehensive subgroup analyses. Finally, the survival outcome analysis was underpowered due to limited access to tissues from patients previously treated with immunotherapy, resulting in an inability to assess the impact of BCAM expression on immunotherapy outcomes. Despite these challenges, we noted a broad range of BCAM expression in various tumor types, including ovarian, lung (NSCLC), HNSCC, bladder, and breast cancers, highlighting its potential as a therapeutic target. The study gains further significance from the comparison with PD-L1 expression. In most cancer types, the lack of correlation between BCAM and PD-L1 expression patterns suggests that BCAM could serve as a therapeutic target for patients less likely to respond to anti-PD-1 and anti-PD-L1 therapies. Additionally, this distinctive expression profile underscores the importance of further investigating BCAM’s role in tumor biology. Understanding BCAM’s unique contribution could offer new insights into cancer progression and the development of innovative therapeutic strategies.

### Supplementary Information

Additional file 1

Additional file 2

## Data Availability

The corresponding authors will provide fluorescent images associated with this study upon reasonable request. Interested parties should contact the corresponding authors directly to discuss data availability and access conditions.
