# Supplementary material for: BCAM (basal cell adhesion molecule) protein expression in different tumor populations
Source: Discov Oncol. 2024 Aug 29;15:381. doi: 10.1007/s12672-024-01244-1 (PMC11362396; doi:10.1007/s12672-024-01244-1)
Supplement: Supplementary file 1 — Additional file 1 [file 12672_2024_1244_MOESM1_ESM.pptx]

## Slide 1
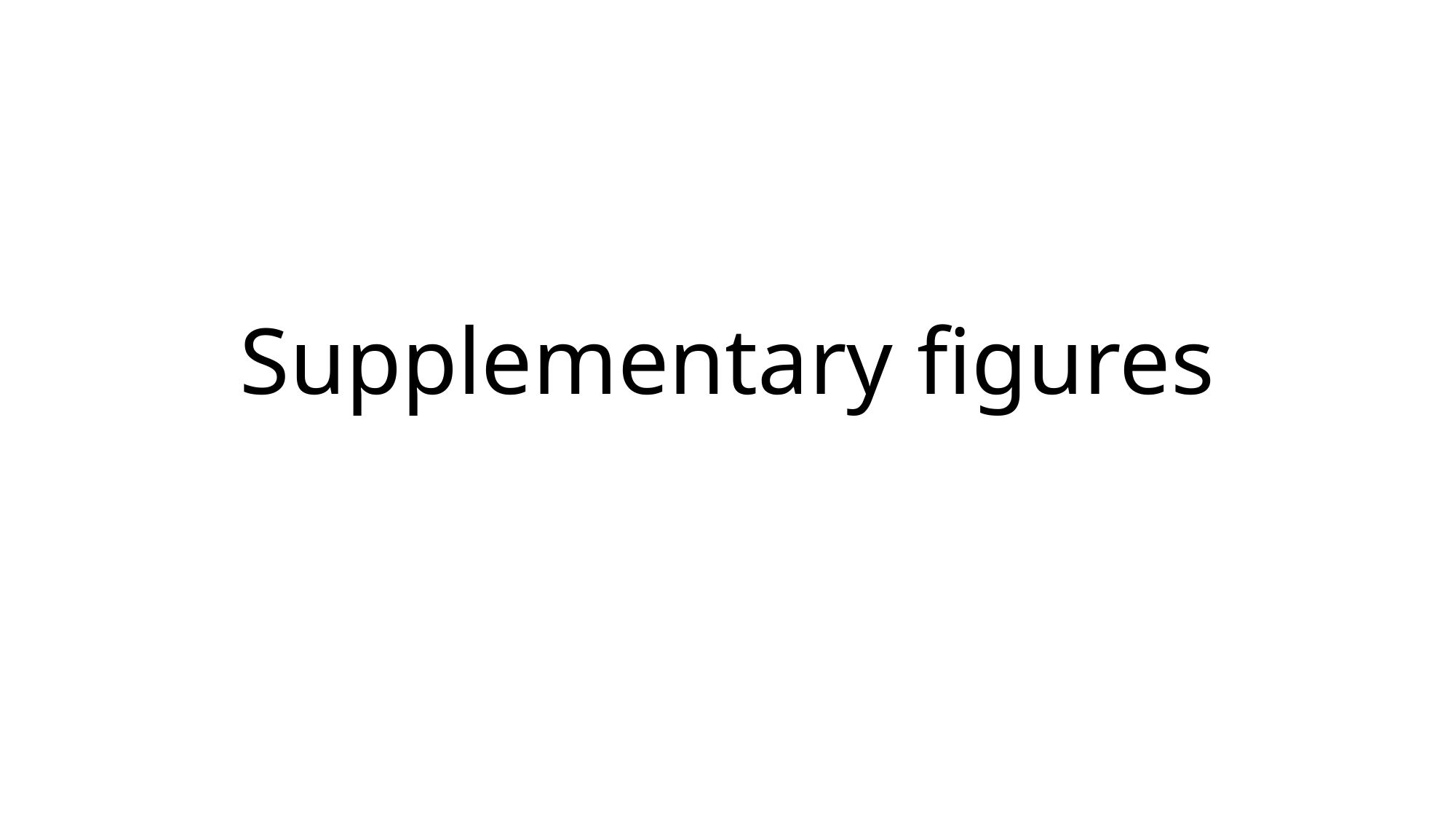

# Supplementary figures

## Slide 2
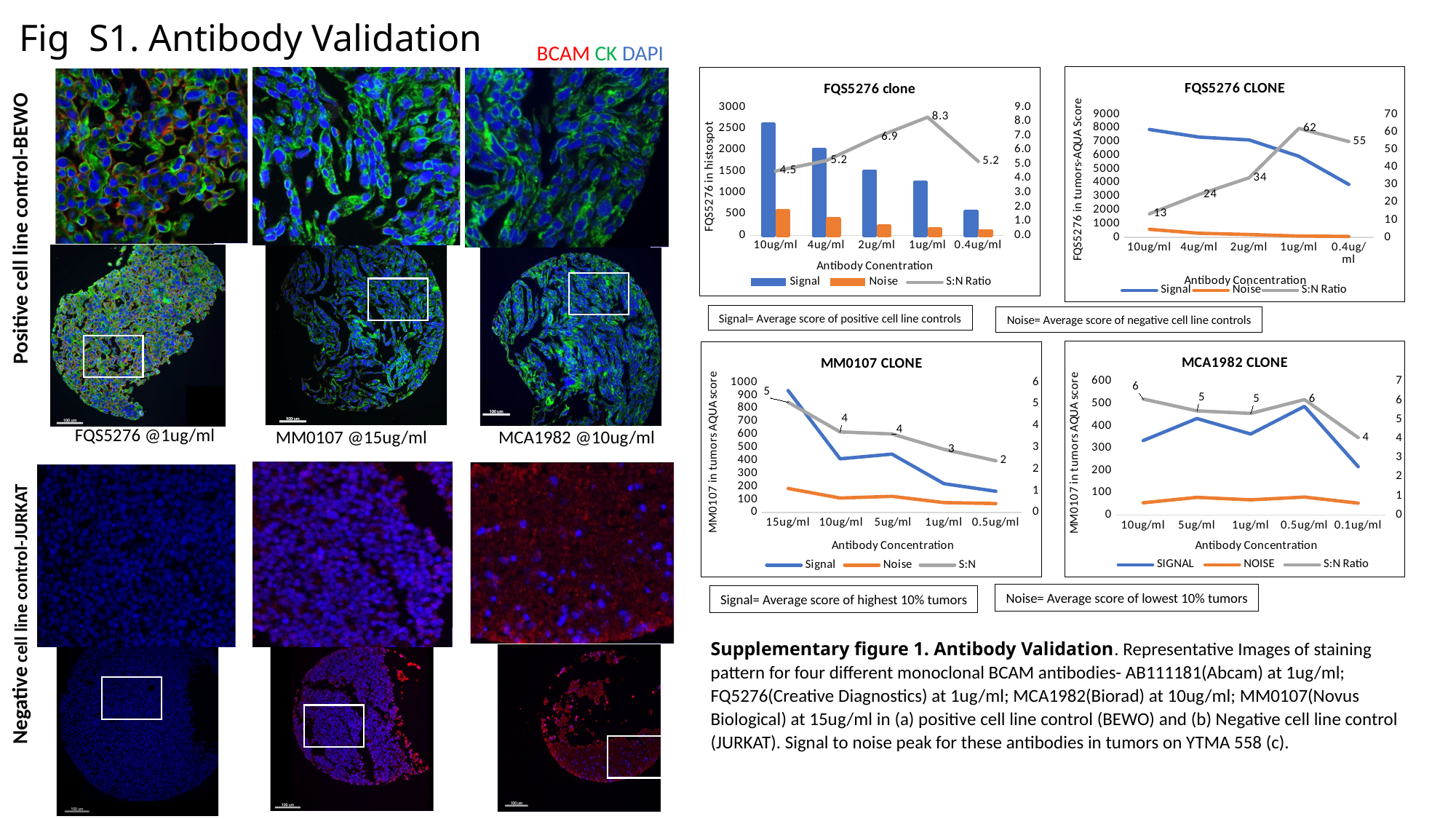

# Fig S1. Antibody Validation
BCAM CK DAPI
### Chart: FQS5276 CLONE
| Category | Signal | Noise | S:N Ratio |
|---|---|---|---|
| 10ug/ml | 7899.334500000001 | 589.8225500000001 | 13.39273057634029 |
| 4ug/ml | 7343.984 | 300.190775 | 24.464389353736806 |
| 2ug/ml | 7135.05925 | 210.100475 | 33.960224268888496 |
| 1ug/ml | 5937.63825 | 95.68566 | 62.05358514536034 |
| 0.4ug/ml | 3876.85225 | 71.0445675 | 54.56929905302049 |
### Chart: FQS5276 clone
| Category | Signal | Noise | S:N Ratio |
|---|---|---|---|
| 10ug/ml | 2598.936833333333 | 576.2551666666667 | 4.510045173853827 |
| 4ug/ml | 2011.2878666666668 | 383.6755333333333 | 5.242158261155738 |
| 2ug/ml | 1504.6653833333332 | 218.24678333333335 | 6.8943301722583605 |
| 1ug/ml | 1241.5799333333334 | 149.83450000000002 | 8.286342153064437 |
| 0.4ug/ml | 558.5745000000001 | 107.67734333333333 | 5.187484039895364 |
Positive cell line control-BEWO
Signal= Average score of positive cell line controls
Noise= Average score of negative cell line controls
### Chart: MCA1982 CLONE
| Category | SIGNAL | NOISE | S:N Ratio |
|---|---|---|---|
| 10ug/ml | 334.45 | 55.02 | 6.0786986550345325 |
| 5ug/ml | 433.47 | 79.42 | 5.457945101989424 |
| 1ug/ml | 364.0 | 68.38 | 5.32319391634981 |
| 0.5ug/ml | 488.0 | 80.68 | 6.048587010411501 |
| 0.1ug/ml | 217.2 | 53.58 | 4.0537513997760355 |
### Chart: MM0107 CLONE
| Category | Signal | Noise | S:N |
|---|---|---|---|
| 15ug/ml | 936.93 | 184.34 | 5.082619073451231 |
| 10ug/ml | 412.5 | 111.05 | 3.7145429986492573 |
| 5ug/ml | 448.47 | 123.91 | 3.6193204745379712 |
| 1ug/ml | 221.31 | 75.98 | 2.912740194788102 |
| 0.5ug/ml | 163.08 | 68.45 | 2.3824689554419285 |FQS5276 @1ug/ml
MM0107 @15ug/ml
MCA1982 @10ug/ml
Noise= Average score of lowest 10% tumors
Signal= Average score of highest 10% tumors
Negative cell line control-JURKAT
Supplementary figure 1. Antibody Validation. Representative Images of staining pattern for four different monoclonal BCAM antibodies- AB111181(Abcam) at 1ug/ml; FQ5276(Creative Diagnostics) at 1ug/ml; MCA1982(Biorad) at 10ug/ml; MM0107(Novus Biological) at 15ug/ml in (a) positive cell line control (BEWO) and (b) Negative cell line control (JURKAT). Signal to noise peak for these antibodies in tumors on YTMA 558 (c).

## Slide 3
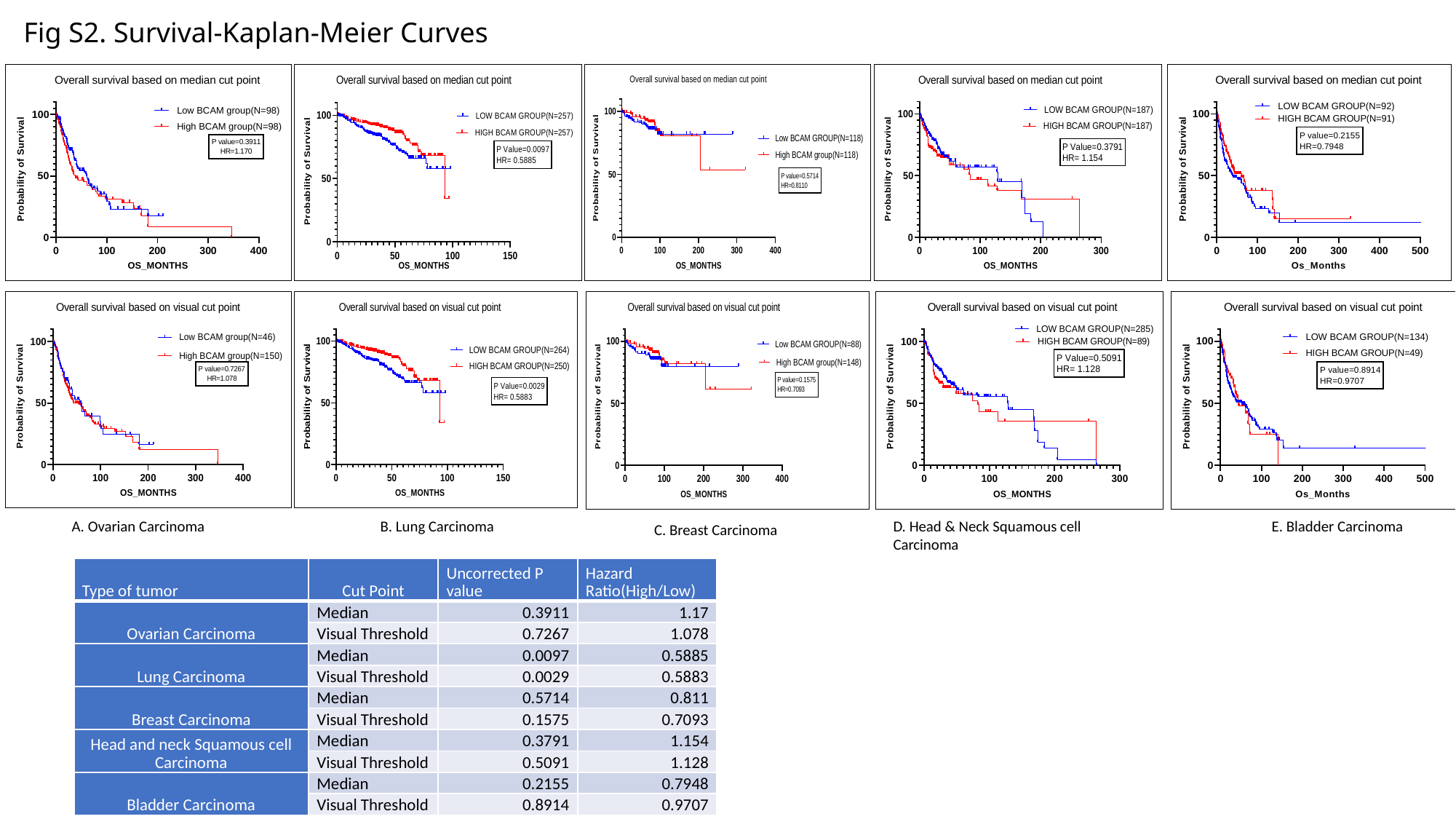

# Fig S2. Survival-Kaplan-Meier Curves
A. Ovarian Carcinoma
D. Head & Neck Squamous cell Carcinoma
E. Bladder Carcinoma
B. Lung Carcinoma
C. Breast Carcinoma
| Type of tumor | Cut Point | Uncorrected P value | Hazard Ratio(High/Low) |
| --- | --- | --- | --- |
| Ovarian Carcinoma | Median | 0.3911 | 1.17 |
| | Visual Threshold | 0.7267 | 1.078 |
| Lung Carcinoma | Median | 0.0097 | 0.5885 |
| | Visual Threshold | 0.0029 | 0.5883 |
| Breast Carcinoma | Median | 0.5714 | 0.811 |
| | Visual Threshold | 0.1575 | 0.7093 |
| Head and neck Squamous cell Carcinoma | Median | 0.3791 | 1.154 |
| | Visual Threshold | 0.5091 | 1.128 |
| Bladder Carcinoma | Median | 0.2155 | 0.7948 |
| | Visual Threshold | 0.8914 | 0.9707 |
