# Supplementary material for: BCAM (basal cell adhesion molecule) protein expression in different tumor populations
Source: Discov Oncol. 2024 Aug 29;15:381. doi: 10.1007/s12672-024-01244-1 (PMC11362396; doi:10.1007/s12672-024-01244-1)
Supplement: Supplementary file 2 — Additional file 2 [file 12672_2024_1244_MOESM2_ESM.docx]

Table 1. Tumor types/Cohorts studied

| **ARRAY TYPE** | **OVARIAN CANCER** | | **LUNG CANCER** | | **BREAST CANCER** | | **COLORECTAL CANCER** | | **BLADDER CANCER** | | **PANCREATIC CANER** | **HNSCC** | |
| --- | --- | --- | --- | --- | --- | --- | --- | --- | --- | --- | --- | --- | --- |
| **TMA #** | YTMA 69 | YTMA 264 | YTMA  423 | YTMA 553 | YTMA 489 | YTMA 499 | YTMA 410 | YTMA 221 | YTMA 361 | YTMA 497 | YTMA 454 | YTMA 465 | YTMA 579 |
| **YEAR RANGE** | 1996-2003 | 1963-2001 | 2012-2016 | 2017-2020 | 2011-2012 | 2013-2014 | 2009-2017 | 2000-2005 | 2005-2014 | 2015-2019 | 2010-2017 | 1999-2018 | 2019-2021 |
| **TOTAL NO. OF SPOTS** | 378 | 342 | 295 | 296 | 263 | 189 | 170 | 280 | 230 | 60 | 248 | 218 | 256 |
| **TUMOR SPOTS** | 358 | 339 | 287 | 296 | 263 | 189 | 155 | 266 | 230 | 42 | 238 | 204 | 247 |

Table 2. Clinicopathological characteristics of ovarian carcinoma array-YTMA 264

| **Characteristic** | **YTMA 264 (N=335)** |
| --- | --- |
| **Median age, years (range)** | 60 (17-90) |
| **Race** |  |
| White | 268 (80%) |
| Black | 7 (2.09%) |
| Asian | 1 (0.3%) |
| Other | 3 (0.9%) |
| NA | 56 (16.72%) |
| **Stage at diagnosis** |  |
| I | 75 (22.39%) |
| II | 18 (5.38%) |
| III | 140 (41.8%) |
| IV | 12 (3.59%) |
| NA | 90 (26.87%) |
| **Histology** |  |
| Squamous Carcinoma | 7 (2.09%) |
| Adenocarcinoma | 110 (32.84%) |
| Embryonal Carcinoma | 1 (0.3%) |
| Mixed tumors | 13 (3.89%) |
| Carcinoma, NOS | 30 (8.96%) |
| Endometrial carcinoma | 57 (17.02%) |
| Others | 117 (34.93%) |
| **Vital status from last follow-up** |  |
| Alive | 123 (36.72%) |
| Dead | 163 (48.66%) |
| NA | 49 (14.63%) |

Table 3. Clinicopathological characteristics of non-small cell lung carcinoma arrays-YTMA 423 and 553.

| **Characteristic** | **YTMA 423 (N = 287)** | **YTMA 553 (N = 296)** |
| --- | --- | --- |
| **Sex** |  |  |
| Female | 175 (61.0%) | 167 (56.4%) |
| Male | 112 (39.0%) | 128 (43.2 %) |
| NA | 0 (0%) | 1 (0.3%) |
| **Median age, years (range)** | 68 (38–89) | 68 (32–89) |
| **Smoking status** |  |  |
| Never | 40 (13.9%) | 1 (0.3%) |
| Former | 180 (62.7%) | 248 (83.8%) |
| Current | 67 (23.3%) | 45 (15.2%) |
| NA | 0 (0%) | 2 (0.7%) |
| **Driver mutation** |  |  |
| *EGFR* | 0 (0%) | 33 (11.1%) |
| *ALK* | 0 (0%) | 0 (0%) |
| *BRAF* | 0 (0%) | 8 (2.7%) |
| *KRAS* | 0 (0%) | 70 (23.6%) |
| *STK11* | 0 (0%) | 1 (0.3%) |
| *TP53* | 0 (0%) | 2 (0.7%) |
| None | 0 (0%) | 47 (15.9%) |
| NA | 287 (100%) | 137 (46.3%) |
| **PD-L1 tumor expression** |  |  |
| Negative (<1%) | 143 (48.8%) | 98 (33.1%) |
| 1–49% | 52 (18.1%) | 57 (19.3%) |
| ≥ 50% | 37 (12.9%) | 34 (11.5%) |
| NA | 55 (19.2%) | 107 (36.1%) |
| **Stage at diagnosis** |  |  |
| I | 200 (69.7%) | 214 (72.3%) |
| II | 66 (23.0%) | 47 (15.9%) |
| III | 17 (5.9%) | 23 (7.8%) |
| IV | 1 (0.3%) | 1 (0.3%) |
| NA | 3 (1.0%) | 11 (3.7%) |
| **Histology** |  |  |
| Adenocarcinoma | 206 (71.8%) | 221 (74.7%) |
| Squamous cell | 67 (23.3%) | 53 (17.9%) |
| Adenosquamous | 1 (0.3%) | 0 (0%) |
| Carcinoid | 0 (0%) | 12 (4.1%) |
| Large cell | 7 (2.4%) | 1 (0.3%) |
| Other/NOS | 6 (2.1%) | 9 (3.0%) |
| **Outcome from last follow-up** |  |  |
| No progression/recurrence | 218 (76.0%) | 252 (85.1%) |
| Progression/recurrence | 46 (16.0%) | 39 (13.2%) |
| NA | 23 (8.0%) | 5 (1.7%) |
| **Vital status from last follow-up** |  |  |
| Alive | 212 (73.9%) | 252 (85.1%) |
| Dead | 70 (24.4%) | 40 (13.5%) |
| NA | 5 (1.7%) | 4 (1.4%) |
| **Survival, months (95% CI)** |  |  |
| Median progression/recurrence free survival | 48.0 (41.0–50.0) | 31.1 (28.2–36.0) |
| Median overall survival | 49.5 (46.0–53.0) | 33.0 (29.0–36.3) |

Table 4. Clinicopathological characteristics of breast carcinoma arrays-YTMA 489, 499.

| **Characteristic** | **YTMA 489 (N= 263)** | **YTMA 499 (N = 183)** |
| --- | --- | --- |
| **Sex** |  |  |
| Female | 258 (98.1%) | 181 (98.9%) |
| Male | 3 (1.1%) | 2 (1.09%) |
| NA | 2 (0.8%) | 1 (0.5%) |
| **Median age, years (range)** | 56 (26-92) | 55 (25-90) |
| **Race** |  |  |
| White | 207 (78.7%) | 159 (86.9%) |
| Black | 31 (11.8%) | 8 (4.4%) |
| Asian | 8 (3.0%) | 7 (3.8%) |
| Other | 2 (0.8%) | 5 (2.7%) |
| NA | 15 (5.7%) | 4 (2.2%) |
| **Smoke status** |  |  |
| Never | 122 (46.4%) | 107 (58.5%) |
| Former | 37 (14.1%) | 70 (38.3%) |
| Current | 88 (33.5%) | 5 (2.7%) |
| NA | 16 (6.1%) | 1 (0.5%) |
| **Hormone Receptor status** |  |  |
| **ER** |  |  |
| Positive | 215 (81.7%) | 153 (83.6%) |
| Negative | 36 (13.7%) | 29 (15.8%) |
| NA | 12 (4.6%) | 1 (0.5%) |
| **PR** |  |  |
| Positive | 182 (69.2%) | 140 (76.5%) |
| Negative | 56 (21.3%) | 39 (21.3%) |
| NA | 25 (9.5%) | 4 (2.2%) |
| **HER _2** |  |  |
| Positive | 28 (10.6%) | 19 (10.4%) |
| Negative | 221 (84.0%) | 160 (87.4%) |
| NA | 14 (5.3%) | 4 (2.2%) |
| **HER2_IHC** |  |  |
| 0 | 36 (13.7%) | 62 (33.9%) |
| 1 | 98 (37.3%) | 60 (32.8%) |
| 2 | 73 (27.8%) | 44 (24.0%) |
| 3 | 23 (8.7%) | 7 (3.8%) |
| NA | 33 (12.5%) | 11 (6.0%) |
| **Stage at diagnosis** |  |  |
| I | 89 (33.8%) | 71 (38.8%) |
| II | 100 (38.0%) | 72 (39.3%) |
| III | 38 (14.4%) | 32 (17.5%) |
| IV | 3 (1.1%) | 4 (2.2%) |
| NA | 33 (12.5%) | 3 (1.6%) |
| **Histology** |  |  |
| IDC | 199 (75.7%) | 132 (72.1%) |
| ILC | 40 (15.2%) | 40 (21.9%) |
| IMC | 17 (6.5%) | 8 (4.4%) |
| Metaplastic | 1 (0.4%0 | 0 (0%) |
| NA | 5 (1.9%) | 2 (1.1%) |
| Other/NOS | 2 (0.8%) | 1 (0.5%) |
| **Vital status from last follow-up** |  |  |
| Alive | 223 (84.8%0 | 164 (89.6%) |
| Dead | 35 (13.3%) | 18 (9.8%) |
| NA | 5 (1.9%) | 1 (0.5%) |

Table 5. Clinicopathological characteristics of colorectal carcinoma arrays-YTMA 221 and 410.

| **Characteristic** | **YTMA 221 (N=254)** | **YTMA 410 (N=151)** |
| --- | --- | --- |
| **Sex** |  |  |
| Female | 133 (52.3%) | 73 (48.3%) |
| Male | 114 (44.8%) | 78 (51.6 %) |
| **Median age, years (range)** | 69.5 (30-97) | 67.2 (28-92) |
| **Smoke status** |  |  |
| Never | 102 (40.1%) | 1 (0.3%) |
| Former | 87 (34.2%) | 248 (83.8%) |
| Current | 25 (9.8%) | 45 (15.2%) |
| NA | 40 (15.7%) | 2 (0.7%) |
| **Race** |  |  |
| White | 207 (81.4%) | 131 (86.7%) |
| Black | 25 (9.8%) | 9 (5.9%) |
| Asian | 2 (0.7%) | 6 (3.9%) |
| Hispanic/spanish | 9 (3.5%) | 5 (3.3%) |
| NA | 7 (2.7%) |  |
| **Stage at diagnosis** |  |  |
| I | 42 (16.5%) | 27 (17.8%) |
| II | 62 (24.4%) | 59 (39.0%) |
| III | 94 (37.0%) | 50 (33.1%) |
| IV | 35 (13.7%) | 13 (8.6%) |
| NA | 21 (8.2%) | 2 (1.3%) |
| **Histology** |  |  |
| Adenocarcinoma | 204 (80.3%) | 129 (85.4%) |
| Mucinous Adenocarcinoma | 28 (11.0%) | 18 (7.0%) |
| Medullary Adenocarcinoma | 1 (0.3%) | 3 (1.1%) |
| Others | 21 (8.2%) | 1 (0.3%) |
| **Vital status from last follow-up** |  |  |
| Alive | 126 (49.6%) | 99 (65.5%) |
| Dead | 107 (42.1%) | 51 (33.7%) |
| NA | 21 (8.2%) | 1 (0.3%) |

Table 6. Clinicopathological characteristics of bladder carcinoma arrays-YTMA 361.

| **Characteristic** | **YTMA 361 (N = 229)** |
| --- | --- |
| **Sex** |  |
| Female | 55 (24.0%) |
| Male | 171 (74.7%) |
| NA | 3 (1.3%) |
| **Median age, years (range)** | 68 (23-92) |
| **Race** |  |
| White | 202 (88.2%) |
| Black | 11 (4.8%) |
| Asian | 3 (1.3%) |
| Other | 5 (2.2%) |
| NA | 8 (3.5%) |
| **Smoke status** |  |
| Never | 147 (64.2%) |
| Former | 22 (9.6%) |
| Current | 8 (3.5%) |
| NA | 52 (22.7%) |
| **Stage at diagnosis** |  |
| I | 46 (20.1%) |
| II | 56 (24.5%) |
| III | 30 (13.1%) |
| IV | 43 (18.8%) |
| NA | 54 (23.6%) |
| **Histology** |  |
| Urothelial | 186 (81.2%) |
| Transitional cell | 23 (10.0%) |
| Squamous cell | 9 (3.9%) |
| Other/NOS | 11 (4.8%) |
| **Vital status from last follow-up** |  |
| Alive | 83 (36.2%) |
| Dead | 135 (59.0%) |
| NA | 11 (4.8%) |

Table 7. Clinicopathological characteristics of head and neck squamous cell carcinoma arrays-YTMA 465 and 579.

| **Characteristic** | **YTMA 465 (N = 103)** | **YTMA 579(N=246)** |
| --- | --- | --- |
| **Sex** |  |  |
| Female | 25 (24.3%) | 75 (30.5%) |
| Male | 78 (75.7%) | 171 (69.5%) |
|  |  |  |
| **Median age, years (range)** | 61 (35-88) | 66(28-93) |
| **Race** |  |  |
| White | 93 (90.3%) | 215 (87.4%) |
| Black | 4 (3.9%) | 9 (3.7%) |
| Asian | 0 (0.0%) | 7 (2.8%) |
| Other | 5 (4.9%) | 11 (4.5%) |
| NA | 1 (1.0%) | 4 (1.6%) |
| **Smoking status** |  |  |
| Never | 20 (19.4%) | 85 (34.6%) |
| Former | 60 (58.3%) | 137 (55.7%) |
| Current | 19 (18.4%) | 22 (8.9%) |
| NA | 4 (3.9%) | 2 (0.8%) |
| **p16 Status** |  |  |
| Positive | 85 (82.5%) | 47 (19.1%) |
| Negative | 11 (10.7%) | 110 (44.7%) |
| NA | 7 (6.8%) | 89 (36.2%) |
| **Stage at diagnosis** |  |  |
| I | 3 (2.9%) | 37 (15.0%) |
| II | 11 (10.7%) | 32 (13.0%) |
| III | 8 (7.8%) | 47 (19.1%) |
| IV | 72 (69.9%) | 101 (41.1%) |
| NA | 9 (8.7%) | 29 (11.8%) |
| **Outcome from last follow-up** |  |  |
| No progression/recurrence | 49 (47.6%) | 97 (39.4%) |
| Progression/recurrence | 50 (48.5%) | 59 (24.0%) |
| NA | 4 (3.9%) | 90 (36.6%) |
| **Vital status from last follow-up** |  |  |
| **Alive** | 49 (47.6%) | 174 (70.4%) |
| **Dead** | 50 (48.5%) | 70 (28.5%) |
| **NA** | 4 (3.9%) | 2 (0.8%) |

Table 8. Antibody details and staining protocol

| **BCAM/CD239** | **HPA005654** | **AB111181** | **MCA1982** | **MM01017** |
| --- | --- | --- | --- | --- |
| CLONALITY | Polyclonal | Monoclonal | | |
| COMPANY | Sigma-Aldrich | AbCam | Biorad | Novus Biologicals |
| HOST SPECIES/Ig | Rabbit | Rabbit Ig G | Mouse IgG 2b | Mouse IgG1 |
| ANTIGEN RETRIEVAL | Citrate buffer @ pH 6 | | | |
| INCUBATION | 1hour at room temp. | | | |
| PRIMARY ANTIBODY | Anti CD239 Rabbit + Mouse CK (AE1/AE3) | | Anti CD239 Mouse + Rabbit CK (polyclonal) | |
| SECONDARY ANTIBODY | Anti- Rabbit envision(HRP) + Alexa 546- GAM | | Anti- Mouse envision(HRP) + Alexa 546 -GAR | |
| TYRAMIDE | Cy5 | | | |
| STOCK CONCENTRATION | 0.10mg/ml | 0. 035mg/ml | 1.0mg/ml | 0.5mg/ml |
| CONCENTRATIONS STUDIED (1µg/ml) | 5, 2, 1, 0.5, 0.1 | 5, 2.5, 1, 0.5, 0.1 | 10, 5, 1, 0.5, 0.1 | 15, 10, 5, 1, 0.5 |
| DILUTION STUDIED | 1:20, 1:50, 1:100, 1:200, 1:1000 | 1:7, 1:14, 1:35, 1:70, 1:350 | 1:100, 1:200, 1:1000, 1:2000, 1:10000 | 1:33, 1:50, 1:100, 1:200, 1:1000 |

Table 9. Antibody details and staining protocol for PD-L1

| **PD-L1** | **E1L3N** |
| --- | --- |
| CLONALITY | Monoclonal |
| COMPANY | Cell-Signaling |
| HOST SPECIES/Ig | Rabbit |
| ANTIGEN RETRIEVAL | EDTA buffer @ pH 8 |
| INCUBATION | 1hour at room temp. |
| PRIMARY ANTIBODY | Anti PD-L1 Rabbit + Mouse CK (AE1/AE3) |
| SECONDARY ANTIBODY | Anti- Rabbit envision (HRP) + Alexa 546- GAM |
| TYRAMIDE | Cy5 |
| CONCENTRATION STUDIED (1µg/ml) | 1.1 |
